# The resource group method in severe mental illness: study protocol for a randomized controlled trial and a qualitative multiple case study

**DOI:** 10.1186/s13033-019-0270-2

**Published:** 2019-03-22

**Authors:** Cathelijn D. Tjaden, Cornelis L. Mulder, Jaap van Weeghel, Philippe Delespaul, Rene Keet, Stynke Castelein, Jenny Boumans, Eva Leeman, Ulf Malm, Hans Kroon

**Affiliations:** 1 Department of Reintegration and Community Care, Trimbos Institute, Utrecht, The Netherlands; 2 Department of Psychiatry, Erasmus Medical Center, Rotterdam, The Netherlands; 3 Antes, Parnassia Psychiatric Institute, Rotterdam, The Netherlands; 4 Department of Social and Behavioral Sciences, Tranzo Scientific Center for Care and Welfare, Tilburg University, Tilburg, The Netherlands; 5 Phrenos Centre of Expertise, Utrecht, The Netherlands; 6 School of Mental Health and NeuroSciences, Maastricht University, Maastricht, The Netherlands; 7 Mondriaan Mental Health Trust, Maastricht/Heerlen, The Netherlands; 8 Department of Community Mental Health, GGZ Noord-Holland-Noord, Heiloo, The Netherlands; 9 Lentis Research, Lentis Psychiatric Institute, Groningen, The Netherlands; 10 Department of Clinical Psychology and Experimental Psychopathology, University of Groningen, Groningen, The Netherlands; 11 Rob Giel Research Center, University of Groningen, University Medical Center Groningen, Groningen, The Netherlands; 12 Sahlgrenska Academy, University of Gothenburg, Gothenburg, Sweden

**Keywords:** Community mental health, Severe mental illness, Recovery, Empowerment, Family, Family intervention, Care structure, (Flexible) Assertive Community Treatment, Resource group, RACT

## Abstract

**Background:**

The resource group method provides a structure to facilitate patients’ empowerment and recovery processes, and to systematically engage significant others in treatment and care. A patient chooses members of a resource group (RG) that will work together on fulfilling patients’ recovery plan. By adopting shared decision-making processes and stimulating collaboration of different support systems, a broad and continuous support of patients’ chosen goals and wishes is preserved and problem solving and communication skills of the RG members are addressed.

**Objective:**

The objectives of this study are (1) to establish the effectiveness of the RG method in increasing empowerment in patients with severe mental illnesses (SMI) in the Netherlands; (2) to investigate the cost-effectiveness and cost utility of the RG method; and (3) to qualitatively explore its dynamics and processes.

**Methods/design:**

This multisite randomized controlled trial will compare the effects of the RG-method integrated in Flexible Assertive Community Treatment (FACT) (90 patients) with those of standard FACT (90 patients). Baseline assessments and 9-month and 18-month follow-up assessments will be conducted in face-to-face home visits. The primary outcome measure, empowerment, will be assessed using the Netherlands Empowerment List (NEL). The secondary outcomes will be quality of life (MANSA); personal, community and clinical recovery (I.ROC); general, social and community functioning (WHODAS 2.0); general psychopathological signs and symptoms (BSI-18); and societal costs (TiC-P). An economic evaluation of the cost-effectiveness and cost utility of the RG method will also be conducted. A qualitative multiple case-study will be added to collect patients’, RG members’ and professionals’ perspectives by in-depth interviews, observations and focus groups.

**Discussion:**

This trial will be the first to study the effects of the RG method on empowerment in patients with SMI. By combining clinical-effectiveness data with an economic evaluation and in-depth qualitative information from primary stakeholders, it will provide a detailed overview of the RG method as a mean of improving care for patients with SMI.

*Trial registration* The study has been registered in the Dutch Trial Register, identifier: NTR6737, September 2017.

## Introduction

Traditionally, severe mental illnesses (SMI) were seen as chronic diseases with relapsing or deteriorating symptoms and poor prognoses [1, 2]. Recovery was perceived as a medical outcome defined by remission of mental health symptoms [3]. Due to the consumer movement, a new view emerged in psychiatry in the 1990s [4, 5]. Within this view, recovery is conceptualized as a unique, personal and ongoing process of growth that involves learning to live with one’s disability despite the limitations of symptoms, and gradually rebuilding a sense of purpose, agency, and meaning in life [5–7].

This conceptualization of recovery was incorporated within the development of new working models for organizing mental healthcare. One of these models is Flexible Assertive Community Treatment (Flexible ACT) [8] that was established in the Netherlands as a Dutch variant of Assertive Community Treatment (ACT) [9]. Flexible ACT teams deliver services for an entire group of people with SMI in a particular region by adapting a flexible switching system between standard community mental healthcare and an intensive ACT equivalent [8, 10]. This combination of flexibility and continuity of care provides opportunities for combining recovery-oriented care with evidence-based medicine, best practices and integrated community and hospital care.

However, an examination of the model fidelity of FACT teams between 2009 and 2014 showed that support of recovery, rehabilitation and participation was implemented insufficiently [11]. Similar findings were shown by a nationwide survey in 2016, which reported that over 80% of patients with SMI experienced feelings of loneliness, that 40% did not feel that they were part of society, and that only 20% had paid or unpaid employment [12]. Second, although the informal support system is perceived as an important factor in supporting recovery and participation and the effectiveness of involving significant others in SMI care is well established, it has been found that systematic and formal forms of support and contact with family members are seldom achieved [3, 11, 13, 14]. These implementation problems justify the ongoing search for a mental health service that empowers patients with SMI, by stressing their choice and autonomy and by encouraging social connectedness and participation.

A structured method for reinforcing empowerment and social connectedness in mental healthcare is represented by the resource group method. In short, to constitute a resource group (RG), patients nominate significant others from their informal network (such as friends and family) and their formal network (such as a social worker or job-coach). During the frequent RG meetings, the RG discusses patients’ goals and wishes, and jointly determines a recovery plan to achieve them.

The first important characteristic of the RG method is that patients themselves take the lead in any decisions: they nominate the members of their RG, set their recovery goals and determine important aspects of how the RG meetings are designed [15]. Considering these decisions is a crucial factor in patients’ sense of autonomy and sense of ownership of their treatment. Patients are then encouraged to extend this to autonomy and ownership of their illness (such as their ability to cope with symptoms) and regarding other social and community aspects of life. This process of regaining control over one’s life—despite the need for support—is a key concept of empowerment, and is regarded as an important driving force in recovery [16, 17].

The second important characteristic of the RG method is that significant others are systematically engaged in treatment and care [18]. As a patient and his or her significant others form a team together with involved professionals, support in the recovery plan is broadened. Hereby, the fulfillment of a meaningful life and everyday activities is strengthened. In other words, through collaboration—joint discussion of patients’ wishes and needs, and creating space for sharing experiences and emotions—an empowered and supportive social environment can be built to supplement professional care. Having such environment in turn, is assumed to foster resilience and continuity in social and community integration. Improved integration and a feeling of connectedness are seen as facilitators and indicators of recovery [1, 19–21].

Also, it is increasingly recognized that significant others need social support to break isolation and reduce stigma [22, 23]. Moreover, studies investigating experiences with care report that families feel marginalized, uninformed, lack a recognized role and distanced from the care planning process [24–26]. Therefore, a structured and more frequent contact between professionals and significant others would meet with their need to feel more part of the treatment and care. Additionally, professional support and attention to the consequences of the patients’ disease for the personal wellbeing of the important people around the patient, may reduce their burden, increase their sense of security, and improve their own mental health status [13, 27, 28]. Moreover, during the RG meetings all involved professional caregivers from different sectors (e.g., mental health, social affairs, housing and employment) can be invited. In this way, the RG method responds to the need to improve communication between all those involved, pursuing a consistent and collaborative model of integrated care.

In sum, the RG method structures the care and support that is built around patients’ personal choices, wishes and aspirations. It focuses on creating a mental health system that encourages patients to be active, informed and autonomous participants who, by collaborating with their social environment, can develop the support that meets their needs and chosen lifestyle. By systematically engaging patients’ significant others, continuity in support is embedded. Eventually, it is hoped, a resilient, empowered social support system can be created that functions independently of professional resources. As the RG method thus has great potential for promoting the autonomy, empowerment and recovery of patients with SMI, it may bring valuable improvements to standard FACT. The origins of the RG method lay in the Optimal Treatment (OT) model, which integrates biomedical, psychological and social strategies in the management of SMI [29, 30]. It was shown in a meta-analysis of the effectiveness of variations of the OT model for patients with a psychotic disorder (N = 2263, 6 randomized studies, 11 observational studies, follow-up between 12 and 60 months) that participation in the OT model led to clinically significant improvements. Relative to care as usual, it improved functioning (Cohen’s *d *= 0.82), increased well-being (*d *= 0.88) and reduced symptoms (*d* = 0.72) [31]. Similarly, a systematic review of eight RCTs showed that the OT program improved symptoms, functioning and well-being in patients with a psychotic disorder [18]. In Sweden, the “family unit in the community” was regarded as a central element of the OT model, and was further developed as the concept of the “resource group” [32]. To reflect the key role of the RG and to integrate it into the existing mental healthcare programs for patients with SMI, the Swedish OT program was relabeled as Resource Group Assertive Community Treatment (RACT) [33, 34]. In this way, ACT teams [9] were enriched and augmented by resource groups involving patients and their network in clinical case management by shared decision making procedures.

This study is intended to add to the existing research in three ways. First, in the studies included in the meta-analysis and review referred to above, integrated care models related to the RACT program were assessed. However, no study has investigated the specific additional value of the RG method in a head-to-head comparison with FACT. Second, previous studies focused on patients with psychotic disorders. Knowledge is lacking about the effectiveness of the RG method for patients across the entire psychiatric spectrum. The third contribution is intended to provide in-depth understanding of the meaning of the experiences in using the RG method to those involved. Very few qualitative contributions have been conducted. As most focused mainly on the case-managers’ point of view [33], they overlooked the experiences and perspectives of patients, RG members and other professionals. To better understand the RG method and its implementation, we thus intend to conduct exploratory research that analyses its dynamics and meaning from the perspectives of those involved.

To achieve these objectives, this study consists of a randomized controlled trial (RCT) to establish clinical effectiveness, an economic evaluation and a qualitative case study on the dynamics, meaning and implementation of the RG-method. The primary objective of the study is to determine whether the RG method integrated in FACT is more effective in empowering patients with SMI when compared to standard FACT. Secondary objectives consist of the assessment of the RG method in improving quality of life and enhancing social and community functioning; and, in an economic evaluation, to investigate its cost-effectiveness. An add-on qualitative study will explore the perspectives of those involved and the implementation of the RG method in Dutch mental healthcare.

## Methods

This three-part study will consist of an effectiveness study, an economic evaluation and a qualitative case study. The study protocol was written in accordance with the CONSORT guidelines [35].

### Part one: effectiveness study

#### Study design

**Fig. 1:**
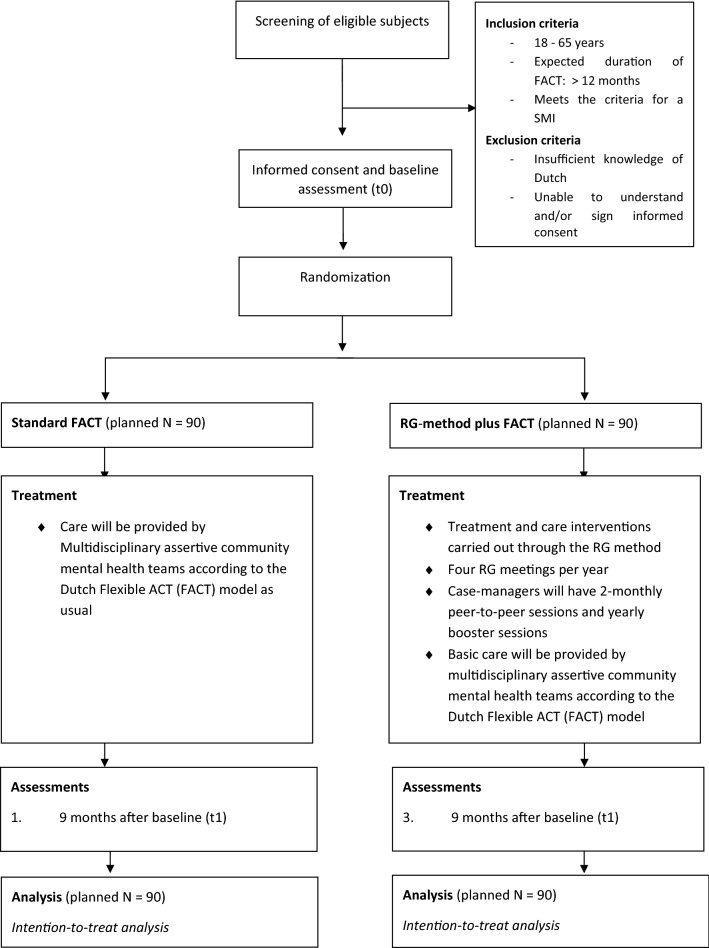
Flow chart of study design. *FACT* Flexible Assertive Community Treatment, *RG* resource group

Patients in this multisite RCT will be randomly allocated either to RG method plus FACT or to standard FACT (ratio 1:1). Randomization will be performed at individual patient level. Data for both conditions will be collected at baseline and after 9 and 18 months (follow-up assessments). For an overview of the flow of screening procedures and assessments, see Fig. 1. Importantly, since FACT teams do almost all outpatient care in the Netherlands for SMI patients, it was not possible to have a second control group without FACT.

#### Study population

The study will be conducted within the context of community-based outpatient psychiatric care for people with SMI. In the Netherlands, FACT [8] is the used service-delivery model for the care and treatment of people with SMI (see “Interventions” for a description of FACT). The target population consists of patients who meet the criteria for the Dutch definition of people with SMI who receive FACT.

##### Inclusion criteria

The inclusion criteria for the study are consistent with the general inclusion criteria for FACT. That is, patients will be eligible if (1) they are aged between 18 and 65 years, (2) are expected to have FACT for > 12 months, and (3) suffer from a SMI according to the Dutch consensus definition [36]. For the latter, individuals must meet the following five criteria, in which:

- They have a psychiatric disorder which requires care and treatment (≈ are not in symptomatic remission);
- They have severe limitations in social and community functioning (≈ are not in functional remission);
- These two criteria are interrelated, with the limitations being the cause and consequence of the psychopathology.
- These problems are not transient in nature (i.e., they are systematic and long-lasting).
- The treatment plan requires coordinated care provided by integrated networks of health practitioners.

##### Exclusion criteria

Patients are not eligible if (1) their knowledge of Dutch is not sufficient for them to understand and read the questionnaires; and if (2) they are unable to understand and sign the informed consent form.

#### Hypotheses and research questions

Hypothesizing that FACT plus RG is a helpful intervention for patients suffering from SMI by improving their empowerment and strengthening their support sources, we state the following research questions:

- Does RG plus FACT increase the empowerment of patients with SMI more effectively than standard FACT?
- Does RG plus FACT improve these patients’ quality of life and satisfaction with care, and enhance their social and community functioning more effectively than standard FACT?

#### Study procedures

##### Recruitment

Patients will be recruited at nine mental healthcare organizations throughout the Netherlands, each of which will participate with minimum two FACT teams. Care providers of the FACT team will screen patients on the basis of the inclusion and exclusion criteria (see Fig. 1). After informing eligible patients of the RG procedures and the study, care providers will then ask them to participate. To this end, patients will receive oral and written information about the RG model and an information letter outlining the trial procedures, explaining confidentiality, and providing the contact details of the research team. Interested patients will be given a week to consider their participation.

The above described procedure for screening and informing patients about the study will be performed on two different groups of patients: either new patients entering a FACT team (i.e., during the intake phase); or a randomly generated selection of patients who have already been in FACT for no more than 24 months. For the latter, we will use an online tool (http://www.randomizer.org), to randomly select patients who have been recently (24 months) added to the caseload of the case-managers trained in the RG method. Importantly, these two routes are used to screen and inform a representative sample of the FACT population of the study. After patients sign informed consent, the researcher will perform an extra check on the inclusion and exclusion criteria and an independent interviewer will contact the participant to make the first appointment for the baseline assessment. After completing the assessment, participants will receive a gift voucher worth €15,-.

##### Randomization and blinding

Randomization will be performed on an individual level. A statistician from the Trimbos Institute, who will be independent from the research team, will perform the randomization using a computer-generated concealed-randomization sequence stratified on teams. To keep randomization unpredictable, the sequence will contain variable-allocation block sizes [37], in which two sizes of allocation blocks (i.e., 2 and 4) are randomized. Assuming ten participants per team, this results in three possibilities for block size 4 (i.e., 2 × 4; 1 × 4; 0 × 4). To minimize the risk of imbalance between conditions, the ratio of these sequences will be stratified on respectively 1:2:2. The allocation sequence will be stored by the independent statistician and be concealed from all researchers, care providers and participants. Participants will be allocated after baseline measurement. Once they have been allocated, the researcher and local staff will be informed of the condition by email. Further matching between patient and case-manager will be performed by the local FACT team staff and will be based on condition. That is, when participants are allocated to the RG condition, care providers trained in the RG procedures will be the case-manager. Patients in the control condition can have any FACT care providers as their case-manager.

Assessments comprise self-report questionnaires, and structured and semi-structured interviews (see Table 3). They will take place at the participants’ homes or any other location they prefer. An independent and blinded interviewer will guide them through the self-report questionnaires and will conduct the interviews. The interviewer will bring a laptop and- using a unique login-code- will assess the questionnaires online, then securing them on an encrypted server (Jambo). If participants are unable to use the laptop, they will fill in the questionnaires on paper and the interviewer will then later enter the data into the online environment.

Given the nature of our study, blinding of participants or care providers is only secured at baseline assessment when condition (e.g., RG + FACT vs. standard FACT) is not known to participants or care providers. However, after baseline assessment condition blinding of participants and care providers is not possible anymore as the condition determines the treatment. Interviewers will be blind for the allocated condition during all three assessments. To assess blinding during follow-up assessment, interviewers will fill in control questions after assessments. To optimize inter-interviewer reliability, interviewers will (1) receive face-to-face training on the study protocol, questionnaires and interviews; (2) discuss the interviewing process with each other in regular telephone and/or face-to-face meetings; and (3) use a detailed standardized study protocol.

#### Interventions

The study will compare two interventions: FACT and FACT + RG. Both interventions will be described below, see Table 1 for an overview of the differences and similarities between the two interventions.

**Table 1:** Overview of the differences and similarities between the two interventions: FACT and FACT + RG

Main elements	Description of FACT	Description of FACT + RG
Involvement of social network	Social network is invited during intake phase and contact can be developed during course of FACT Actions A contact person is established for each patient and contact details are provided Family or significant others can be invited as FACT proceeds In the event of (upcoming) crisis, the contact person is informed	Social network (including family, friends, colleagues and significant others) are structurally involved and collaborate as partners in treatment and goals Actions Within 3 months, nominated significant others from the social network meet the FACT staff for the interview During the RG meeting, the RG members are actively involved in maintaining the goals FACT staff and RG work together as a team (equal experts)
Treatment/recovery plan	Recovery goals are developed by client and caregiver (treatment plan) and are discussed during the FACT meeting To achieve these goals, the FACT team allocates tasks and responsibilities on the basis of expertise The treatment plan is discussed at least once a year by the multidisciplinary FACT team The treatment plan contains SMART formulated, concrete goals	Recovery goals are developed by client and caregiver (RG plan) and are discussed with the RG members (possibly including FACT team members) during the RG meeting The client decides together with the RG on actions to be taken to achieve the goals The RG plan is discussed once every 3 months by the RG; the psychiatrist is present at least once a year The RG plan contains two long-term goals (= future dreams and wishes) and two short-term goals (= SMART formulated, concrete goals)
Continuity of care	FACT contains two modes of operation within the same team: high-level intensity (ACT, adaption of shared caseload) and low-level intensity (Individual Case Management). The flexibility to switch between them enhances continuity of care	Additional to the flexibility in FACT, the flexible composition of the RG incorporates various institutes and people and allows a broader range and intensity of care. Although the RG members may differ, the RG itself is the constant factor

##### Standard Flexible Assertive Community Treatment (FACT) (for a more comprehensive description, see [38])

FACT is a rehabilitation-oriented outpatient clinical case management model for patients with severe mental illness. Integrated care and support is provided in the patients’ own environment by a multidisciplinary team of professionals (e.g., psychiatrist, psychologist, nurses, social worker, job coach and peer specialist). On average, a FACT team consists of 11–12 professionals that monitor 200 patients [38]. The FACT model is characterized by its flexible switching between two types of care, according to patients’ needs:

1. *Individual case management for more stable patients.* The case-manager visits a patient 2–4 times a month at his/her home or elsewhere and is responsible for the individual care and treatment plan. This plan is renewed at least once a year and is formulated in a way that patients and their families can understand. Part of this plan can be a so-called crisis plan, which describes early-warning symptoms and concrete arrangements for intensifying care if necessary. Appointments with the psychiatrist (for management and evaluation of medication) and with the psychologist (for psycho-education or cognitive behavior therapy) can take place at the FACT center or at the patients’ home. On indication, family interventions and supported employment may be added to the treatment plan.
2. *Shared case management and intensive assertive outreach care for unstable patients who are at risk of relapse, neglect or readmission*. The care for the individual patient is intensified but performed by the same team. That is, this group of patients is discussed daily during the team meeting using the digital FACT-board (DigiBoard); the psychiatrists sees the patient within 2 days; the crisis plan is updated and set in motion; and the case-manager informs the patient (and if necessary the family) that more intensive care will be organized and that colleagues from the FACT team will work together to prevent readmission and to shorten the crisis. If the crisis or risk of relapse has decreased and the situation has stabilized, the care is shifted back to individual case management.

To date, the effects of FACT have not been studied in the context of an RCT. Uncontrolled studies have shown a pre-post effect on symptoms and admissions [10, 39–41].

##### Resource group plus FACT

In this condition, patients will be guided to form a resource group (RG) embedded within FACT. In other words, together with the case-manager, patients will prepare, attend and evaluate 3-monthly RG meetings that are integrated in standard FACT.

An essential element in the RG method is the position of the patient as the director of the group [30]. The patient nominates the RG members, determines his or her short- and long-term recovery goals, and decides on the location, chairman and agenda of the RG meetings. As a patient’s ownership of the treatment is vitalized by explicitly thinking about and determining these aspects of care, it is also essential to the patient’s empowerment—which, in turn, was shown to be the major driving force behind successful treatment [18, 31].

*RG members*: The patient will ask his/her significant others to join the RG, a process referred to as nominating. The composition and size of the RG are flexible, and can change over time according to patients’ goals and phase of recovery. The patient and the case-manager always attend the RG meetings. At least once a year, the psychiatrist of the FACT team will attend the RG to evaluate the recovery plan. Before the first RG meeting, the case-manager will invite the nominated RG members for an interview that explores working with the RG and the commitment and responsibility of being a RG member. Also, the relationship between the nominee, the patient and other RG members and previous experiences in good and bad times are investigated. Exploring these emotions and experiences will provide valuable information and will also provide insight into the personal wellbeing and burdening of significant others. Discussing these objectives at an early stage is also intended to reduce the so-called expressed emotions (EE) [42] during the RG meetings. Having individual contact with relatives before initiating any activity involving groups is also considered essential to structured work together [13]. The aim is for all RG members to work together in an emotionally stable environment that contributes to a resilient and continuous support system. Previous experiences with the RG method showed that most of the nominated RG members agreed to participate [33]. However, in some cases the network of a patient might be dysfunctional or almost invisible, or the significant other is unable or does not want to participate. In these cases, the RG will start with the minimal composition of a RG, consisting of the patient, the case manager and the psychiatrist. Together they will work on the steps that the patient or his/her significant others need to expand the RG. By means of the model-fidelity scale we will collect information on the composition of every RG.

*Recovery plan*: To prepare the RG meetings, the patient and case-manager will develop the recovery plan that is to be discussed during the RG meeting. This plan will comprise two long-term recovery goals, two short-term (i.e., 3-month) recovery goals, and a plan to recognize early warning signs (a “crisis plan”). The recovery goals will be formulated by the patient and can relate to all aspects of recovery, such as personal recovery (recovering identity); social rehabilitation (meaningful participation in society and social relationships); and health (improving physical and mental symptoms). In the crisis plan, patients will describe how others should recognize the personal early warning signs that indicate an approaching relapse, and how they want others to respond.

*Resource-group meetings*: RG meetings are usually scheduled once every 3 months, but the frequency may vary according to needs and wishes of the patient and the other RG members. The meetings will be structured clearly and consistently by an agenda that is determined by the recovery plan. The role of each member in accomplishing the recovery goal will be decided jointly by the RG, which will take shared responsibility for following the plan (shared decision making). The patient determines what the overall objectives of the RG meetings should be, and the group takes joint decisions on how they should be achieved [32, 33]. Between meetings, the patient, RG members and care professionals will work on the different parts of the recovery plan, using the next RG meeting to evaluate the steps they have taken. During these activities in between, the empowerment of the patient and the collaboration of the different RG members form the fundamental elements that shape the contact. Also, the crisis plan will be discussed during one of the first RG meetings. In the event of crisis or the need to prevent it, the aim is to enable RG members to provide the effective, adjusted guidance determined by the patient.

Previous experiences have shown that in some cases it might take time to organize an actual RG meeting [33]. Moreover, sometimes there are unsolved issues between RG members that need to be addressed in order to have a constructive meeting with low EE. This could cause a delay in the occurrence of the RG meetings. However, the preparation in which the patient actively takes part in the planning and is involved as a key decision maker is considered to be a crucial factor in the empowerment of the patient [18, 31]. The increased commitment of the case managers to involving the informal support system is also starting in the preparation phase. Hereby, the shift towards empowering the patient, restoring his/her self-confidence and increased attention for the interactions in the informal support system is gradually taking place before and in between the RG meetings.

*RG members’ skills*: As well as the 3-monthly RG meetings, the RG method comprises several options for proving specific training sessions. The case manager and/or other professionals train the patient and the significant others to allow them to improve their skills to communicate, handle stress and solve everyday problems. No costs are involved for the RG members. The need for these training sessions can be addressed by all RG members. In this way, maladaptive patterns and potential stressors in the patient’s environment can be addressed so as to create a healthy and communicational emotional climate around the patient. Alternatively, when more complex problems are evident, the RG members can decide to involve an expert—in family therapy, for example—for an extra session for the complete RG or a subgroup of it.

To set up, structure up and continue a RG in the way described above, the patient and case-manager will jointly pass through six phases. For a description of each phase, see Table 2.

**Table 2:** The six phases of the RG-method

Phase	Actions
Preparation	Patient and case-manager draft sociogram Patient and case-manager nominate RG members Patient and case-manager draft the RG plan (containing two long-term goals; two short-term goals; crisis plan)
Investment	Case-manager establishes contact with nominated significant others Case-manager interviews nominated significant others, covering at minimum Their expectations of, commitment to and responsibility in the RG Their relationship and previous experiences with the patient and other nominated RG members Their contribution to the RG
Planning	Patient and case-manager set date of first RG meeting Patient and case-manager set up and print agenda Patient decides The location of the RG meeting The chairman The frequency of the RG meetings The channel of communication between the different RG meetings
First RG meeting	All RG members introduce themselves or are introduced by the patient The patient and/or case-manager give a short explanation of the RG method and confidentiality The RG discusses the agenda The RG goals The crisis plan The role of each RG member, concrete actions to achieve the RG goals
Follow-up RG meetings	During the follow-up RG meetings The RG evaluates goals, assignments and progress The RG updates the goals and the RG plan, and decides on new actions to achieve the goals Skills trainings are available for RG members (e.g., problem solving and emotional communication) When wished by the patient or another RG member, the composition of the RG can change if different persons are better suited to achieve the updated goals Once a year psychiatrist attends the RG
Reorientation	Discussion on composition of the RG, depending on the phase of care De-intensification of care: transition to GP/social domain or to only informal RG members Intensification of care (e.g., crisis plan)

#### Implementation

To ensure that the RG method is implemented solidly and in a similar fashion across the different centers and teams, an implementation strategy with several components was developed. This strategy involves the following components: (1) training in the RG method for participating case-managers of the FACT team; (2) regular visits by research teams (at least once every 3 months) to ensure good communication; (3) newsletters to keep teams and care providers informed and involved; (4) six-weekly telephonic peer-to-peer meetings among case-managers working with the RG method; and (5) questionnaires after every RG meeting (to ensure model fidelity). Three of these components require a more detailed description:

##### Training in the RG method

At least two members from each FACT team will participate in a 2-day training program before the start of the study, and in 2 follow-up sessions during the study itself. Additional yearly booster sessions will also be organized. Two experienced trainers, one of them being a family therapist, will lead the interactive program. The program will consist of lectures, role-play and discussions that enable case-managers to study and familiarize themselves with the vision, methodology and content of the roles within the RG method. During these days, the central theme will be ensuring that case-managers learn the reflexes necessary to transferring the guidance in treatment to patients and their RG so as to nourish patients’ confidence in reaching their goals. Mental health institutions and teams are selected to participate when they expressed their motivation to be involved in the national effectiveness study and are interested in the implementation of the RG method. Within the participating teams, team members decide between themselves who will be trained. An estimated number of 50 members of the different FACT teams will be trained. Most of them will be working as a case-manager or nurse, and also some peers-by-experience workers, psychiatrists and psychologists will be encouraged to participate to pursue a broad implementation.

##### Model fidelity

The adherence of each RG to the RG protocol will be assessed with a new instrument: the Resourcegroup Model Evaluation Tool (R-MET), which was developed on the basis of the Dutch RG handbook [43], and documents for assessing RG model fidelity developed in Sweden during previous studies. The purpose of the tool is to estimate the extent to which an individual RG operates according to the intended approach. In collaboration with experts by experience, representatives of the participating mental health centers and researchers, the tool was drafted, tested, adjusted, and will be implemented in all teams. To obtain a model-fidelity score, the patient, RG members and case-manager will fill in questions that provide an overall picture of each individual RG. By collecting the answers from the different people that are involved in an individual RG, different perspectives are integrated in the final model-fidelity score.

The R-MET has two sub-forms that together compose the RG model fidelity score. The forms are to be filled in as specified here:

1. *RG meeting form* This form consists of 25 short questions that are filled in by the case-manager in consultation with the patient after each RG meeting. The questionnaire collects information on characteristics of the RG (e.g., its members, chairman and frequency of RG meetings), on its preparation (interviews with nominated RG members and drafting the agenda), the recovery plan and the patient’s degree of ownership. The emotional environment of the group is also assessed. For this, five Visual Analogue Scales (VAS) are used to review the five domains of EE: hostility, emotional over-involvement, critical comments, warmth (reversed), and positive comments (reversed) [42]. Because the questions are filled in after each RG meeting, recurring information on the individual RG is collected. This not only gives insight in the development of the RG but is also a way to keep track of the progress of all RGs.
2. *Yearly evaluation form* This consists of 9 questions and is completed by the patient, RG members and case-manager once every 12 months before a RG meeting. During the RG meeting itself, the RG jointly evaluates the RG meetings by discussing the questions. As well as contributing to model fidelity, filling in this yearly evaluation form thus provides input for optimizing the RG. It uses different VAS to evaluate how the different RG members experience the main features of the RG method. Its themes are the emotional environment with regard to trust, equality, and responsibility during the RG meetings. In addition, the patient fills in some questions on his or her experience of ownership of the RG. Finally, all RG members, including the patient and case-manager, rate satisfaction with the RG meetings.

##### Telephonic peer-to-peer sessions

All trained case-managers attend 6-weekly telephonic peer-to-peer sessions. These are 1-h group sessions that are held by telephone by a fixed group of no more than 8 case-managers from the different mental health organizations throughout the Netherlands that participate in the study. Each group has a chairman, who leads the sessions. To keep track of recurrent themes and of quality across the sessions, a researcher also attends the sessions. During the sessions, case-managers exchange their RG experiences and discuss individual cases, the aim being to learn from each other regarding RG work and to improve the quality of the individual RGs.

#### Outcome measures

Several instruments (questionnaires and interviews) will be used in the clinical effect and economic evaluation studies. See Table 3 for an overview of outcomes and instruments.

**Table 3:** Outcomes and instruments

Measurement	Outcome	Instrument (type of assessment)	Time (min)
Primary	Empowerment	NEL (self-rated)	15
Secondary	Demographic information	DEM_1 (self-rated)	10
	Quality of life	MANSA (self-rated)	5
	Recovery	I.ROC (interview)	15
	Community and social functioning	WHO-DAS 2.0-36 (interview)	15–20
	Global functioning	GAF/SOFAS (observer-rated)	5
	Social contacts	DEM_2 (self-rated)	10
	Clinical symptoms	BSI-18 (self-rated)	5–10
	Attachment	RAAS (self-rated)	5–10
	Satisfaction with care	CSQ, domain relatives involvement VSSS-EU (self-rated)	5
Economic evaluation	Use of healthcare services	TIC-P (interview)	10
	Quality of life	EQ-5D-5L (self-rated)	3
Significant others	Burden of significant others	IEQ (filled in by informal support system)	10

*Baseline demographic information and clinical information (DEM_1)*: Basic demographics (self-report) will be collected, including age, gender, education, housing, country of birth, and children. In addition, data will be collected on the duration of psychiatric illness, on alcohol/drug use, on psychiatric diagnosis and on history of psychiatric care (including the number of voluntary and compulsory admissions).

#### Primary outcome: empowerment

*Netherlands Empowerment List (NEL)*: The NEL (40 items, self-report) contains 6 subscales: confidence and purpose (12 items); social support (7 items); connectedness (6 items); self-management (5 items); caring community (6 items); and professional help (4 items). Items are rated on 5-point Likert scales (strongly disagree–strongly agree). We will use the total score of the NEL as our primary outcome measure. The scale was defined in collaboration with patients and experts-by-experience, and has been validated [17]. Sensitivity to change has been demonstrated [44, 45].

#### Secondary outcomes

##### Quality of life

The Manchester Short Assessment of Quality of Life (MANSA; 16 items, self-report) is a shortened version of the Lancashire Quality of Life Profile (LQLP) [46]. It reliably measures quality of life in patients with psychological problems [47].

##### Recovery

The Individual Recovery Outcomes Counter (I.ROC; 12 items, interview) has been found to be a valid and reliable measure of recovery in mental health [48].

##### Social and community functioning

To obtain information regarding social and community functioning, several self-report questions (DEM_2) on education, work, social network and frequency and quality of social contact will be included.

##### Role functioning

The World Health Organization Disability Assessment Schedule-36 items (WHODAS 2.0-36, interview) produces reliable disability measures across six domains to assess general, social and community functioning [49–51].

##### Global functioning

The global assessment of functioning scale (GAF) and the social and occupational functioning scale (SOFAS) will be derived from DSM Axis V to assess global functioning and symptom severity (GAF) and social functioning (SOFAS) [52–55]. The interviewer, who is blind for condition, will administer both scales after each measurement.

##### Clinical symptoms

The Brief Symptom Inventory-18 items (BSI-18; self-report) is a validated, reliable instrument for assessing general psychopathological symptoms as an index of severity of syndromal disorders [56–58]. As well as the total score, a dimensional score on somatic complaints, depression and anxiety will be obtained.

##### Attachment

The Revised Adult Attachment Scale (RAAS, 18 items, self-report) has moderate to good psychometric properties for assessing attachment style [59–61].

##### Satisfaction with care

Patients’ appreciation of care will be assessed using the Client Satisfaction Questionnaire (CSQ-8) [62] supplemented with the relative’s involvement dimension of the Verona Service Satisfaction Scale (VSSS-EU) [63]. The CSQ-8 is a one-dimensional 8-item instrument for assessing global patient satisfaction. It has demonstrated high construct validity and internal consistency reliability [64], also in Dutch [65]. The relative’s involvement dimension of the VSSS-EU consists of six items that cover various aspects of the patient’s satisfaction with help given to his/her closest relative. Also, four self-formulated questions were added, two covering the degree of patients’ satisfaction with the role of their relatives in their treatment, and two covering patients’ satisfaction with the collaboration of the various services involved.

#### Cost-effectiveness

##### Cost data and quality of life

The Trimbos and Institute of Medical Technology Assessment Cost Questionnaire for Psychiatry (TIC-P, interview) estimates use of care services, use of medication, and the amount of work loss (absenteeism and reduced efficiency) [66]. The questionnaire will be adapted to fit the purpose of this study and uses a 3-month recall period.

The 5-level EuroQol 5 dimensions (EQ-5D-5L) is a standardized non-disease-specific instrument that will be used to obtain utility scores on the basis of social tariffs, expressed in Dutch unit prices [67, 68].

#### Significant others

##### Burden of significant others (filled in by significant others)

The Involvement Evaluation Questionnaire (IEQ, 31 items, self-report) assesses the consequences of mental illness for significant others [69, 70]. It will be sent online to the significant others who are proposed by the patient.

#### Sample size

Power calculations of the study will be based on the earlier described meta-analysis investigating the effectiveness of RACT [31]. Using the program G*power (two-sided, power = 80%, alpha = 0.05; G*power 3.1) with a medium effect size (*d* = 0.5) [31], we found that a total of 126 participants would be sufficient to detect a statistically significant difference between the two conditions. If account is taken both of repeated measures within a person (assuming a within-correlation of 0.6) and of clustering of data (teams; assuming an intraclass correlation coefficient (ICC) of 0.05, health centers; assuming an ICC of 0.1), a sample of N = 133 is needed. To account for possible drop-outs (rate 35%), we aim to recruit a total sample of N = 180. Eighteen teams at a total of nine Dutch mental health centers will participate, each with two teams. In principle, each team should deliver a mean of N = 10 patients per team over the course of 1 year.

#### Analyses

Outcome data will be analyzed using multilevel mixed regression models with 4 levels: observations within people, people within teams, and teams within centers. Analyses will be conducted on the entire randomized sample (i.e., intention to treat). Supplementary analysis will be done on the completers sample. A completer will be defined after further inspection of the frequency of the RG meetings to define a minimum of attendance of a RG meeting during a 12-month period after the first assessment. In future publications the number of minimum RG meetings will be clearly stated within the definition of a completer and included in the consort flow chart of the RCT. To analyze potential between-condition differences in baseline characteristics (such as gender and diagnosis), we will use Student’s t-tests for continuous variables and Pearson Chi-squared tests for categorical variables. As a covariate, the analysis will include variables that show different distributions in the conditions (p ≥ 0.05 difference at baseline) and are correlated with the results. For categorical outcome variables we will choose counts and, if there are non-normal residuals, appropriate forms of mixed regression (such as binomial, Poisson and gamma). All analyses will be carried out using SPSS version 20+ and/or R version 3.0+. Results will be described in accordance with the CONSORT guidelines for randomized controlled trials [35].

### Part two: economic evaluation

The economic evaluation will involve both a cost-effectiveness analysis (CEA) and a cost-utility analysis (CUA). It will be performed from a societal perspective according to the intention-to-treat principle, using imputation to address missing data on the basis of the latest guideline for health-economic evaluation [71, 72]. All costs will be expressed in euro. Costs will be divided into one of three types: (1) main intervention costs in participating healthcare center; (2) mental healthcare utilization (e.g., medication, general practitioner, emergency care, outpatient visits to a general hospital, housing counseling, and admissions to a general hospital); and (3) costs stemming from productivity losses in paid work and volunteer jobs (both due to absenteeism and less efficiency while at work). Costs and outcomes will be evaluated at baseline, 9 and 18 months (parallel with the randomized trial).

#### Research question

- From a societal perspective, is the addition of RGs to FACT preferable to FACT alone in terms of costs, effects and utilities?

#### Analysis

At baseline, the homogeneity of groups will be assessed with regard to both costs and outcomes. Where necessary, we will control for baseline differences [73, 74]. The primary outcome parameter for the CEA will be treatment response after 18 months, which is defined as within-patient pre-post increase in empowerment (NEL). For the CUA, we will convert the health states resulting from the five dimensions of the EQ-5D-5L into utilities based on the Dutch tariffs of the EuroQol, the so-called EQ-5D value set [68]. Using the area under the curve (AUC) method, the periods between the assessments will be weighted by these computed utilities. This will allow quality-adjusted life years (QALYs) to be adjusted over the entire trial period. Similarly, cumulative costs over the entire follow-up period will be obtained from the cost estimates at the various assessments [67, 75]. The total QALYs gained during 18 months is the primary outcome of the CUA.

Incremental cost effectiveness ratios (ICERs) will be calculated for both CEA and CUA: ICER = (C1 − C2)/(E1 − E2), where C refer to costs, E to effects, and subscripts (1 and 2) to the two trial conditions (RG + FACT/standard FACT). These ICERs express the average incremental costs associated with 1 additional unit of the measure of effect [76]. For the CEA, this refers to the incremental costs per treatment responder (= increase at the NEL); for the CUA, it is the incremental costs per QALY gained. Next, confidence intervals around the ICER will be computed using a nonparametric bootstrap approach: > 2500 non-parametric bootstrapped samples will be extracted from the original dataset. For each of the bootstrapped samples, the incremental costs, incremental effects, and the incremental cost-effectiveness ratio (ICER) will be calculated. The point estimates of the mean ICER and the resulting > 2500 ICERs will be used for further calculation, and will be graphically displayed in a cost-effectiveness plane [76]. Sensitivity analyses will be performed to assess the robustness of the findings. When conducting the analyses and describing the results, we will follow the CHEERS guideline for health-economic evaluations [77].

### Part three: qualitative case study

The qualitative case study will be performed to improve our understanding of the RG method. It will focus on the dynamics of the RG, its meaning to those involved, and conditions for successful implementation. To this end, a multiple grounded case study with an interpretative, inductive analysis will be carried out [78–80]. To increase validity, two people will jointly perform the case studies.

For inclusion, patients will be selected by means of the information derived from the baseline measurements taken during the quantitative study. Selected patients will then be approached for their approval for participation and to sign informed consent. Variation in inclusion will be pursued in terms of the time patients have been in care, the size and composition of the RG and the therapeutic working relationship. The case selection takes place in several steps. Based on the experiences with the first cases, new cases will be selected. We expect to include a total of approximately 6–8 patients and their RG before saturation occurs, saturation being the point at which sampling more data will not produce more information on the emerging theory and research question, or greater insight into them [78]. The aim of this so-called purposive sampling is to produce a sample that can be assumed to be representative of the variety of the population.

Over the first year, the progress of all included patients and their RG will be followed closely. To this end, interviews will be held with the patient at several time points, and various parts of the process will be observed, such as goal-setting, the RG meetings and their evaluation. To complement patients’ view, interviews will be also held with different stakeholders (RG members and professionals). Participation in this part of the study will be voluntary, and will also take place independently of participation in the quantitative part. In a member check, all participants—thus patients and RG members alike—will be invited to attend a focus group session in which the main outcomes of the interviews and observations are discussed. They will be asked to verify whether their opinion has been expressed correctly.

#### Research questions

- How do RG dynamics develop in practice?
- What is the significance to patients and the other RG members of participating in the RG?
- How and under what circumstances can the RG influence a patient’s personal processes of recovery?
- How and under what circumstances can the RG influence the resilience of the social network?

#### Analysis

To provide scope for exploring any unexpected aspects of the material the analysis and data collection will be interwoven [80]. The analysis will be performed according to the constant comparative method, in which, to develop the theory as it emerges, two analysts jointly collect, code and analyze the data, deciding as they go which data to collect next [80]. To generate theories iteratively, we will also perform three rounds of coding: initial coding, focused coding and theoretical coding [78]. To investigate unique processes within individual RGs, and the similarities and differences with other RGs, “within case” and “cross-case” analyses will be performed. To code and compare text fragments, themes, and concepts, we will use software for qualitative analysis (MAXQDA).

## Discussion

This paper describes the study protocol for assessing the effectiveness, cost-effectiveness, meaning and implementation of the RG method for patients with severe mental illnesses. Our primary outcome measure is the empowerment of the patient in the RG.

This study has the potential to address two key issues in the care for patients with SMI. First, by combining clinical-effectiveness data with an economic evaluation and in-depth information from primary stakeholders, it will provide a thorough overview of the potential of the RG method to improve mental healthcare for patients with SMI. Giving patients directorship and systematically involving significant others both represent a break with more traditional forms of treatment, as they change the dynamics between patients, professionals and significant others. Using mixed methods to investigate the consequences will provide profound insights into the working mechanisms of the method, and will allow a clear prescription for the implementation of the RG method in Dutch mental healthcare.

Second, even though significant others are in principle supposed to be involved within FACT, formal forms of integrating family into FACT are absent or limited in practice [11]. The RG method fills this gap because it not only engages and activates resources of the informal network, it also pays attention to the subjective wellbeing, psycho-education knowledge and mutual communication- and problem solving skills of patient’s significant others. As well as having the potential to form a broad and stable social and community integration, the method hereby also contributes to a resilient emotional social environment.

Some potential risks for bias are to be expected. First, although efforts are made to include the full range of severely mentally ill patients from the FACT population, it may still prove difficult to include patients who are not motivated to involve their social network within mental healthcare. This means that great caution will be necessary when generalizing the results to all patients in FACT-care—including those who have a difficult or non-existent relationship with their social network. In any case, generalization will be possible only after thorough inspection of the data and baseline data.

Second, in line with the RG model, patients will decide who will be nominated as RG members. This may mean that they do not select people from their informal support system (e.g., family, friends, colleagues), but only from their formal support system (e.g., professionals from within and/or outside mental healthcare). However, previous studies indicate that the variety in the RG composition and the engagement of the informal support system might be determining factors in the effectiveness of the RG method [31]. It is therefore possible that potentially positive effects are missed because the informal environment is not engaged. However, as the main intention of the RG method is to develop agency over and ownership of treatment, it would conflict with the model if patients were obliged to include certain people. To deepen understanding of the effect of engaging the informal support system within the RG, the qualitative case study will seek to include cases with varying RG compositions (e.g., with and without informal support system).

Third, because the same FACT team will perform treatment and care for both conditions, it is possible that elements of the RG method will spill over into the standard FACT control condition. Although trained caregivers will be explicitly instructed not to integrate aspects of the RG method within the standard FACT condition, it cannot be ruled out that discussing and thinking about the RG method will lead to the unconscious application of principles of the RG method within standard FACT.

Fourth, the RG method has a specific structure, and identifies clear steps for putting the intended philosophy in practice. As such steps are not described so clearly within standard FACT, there is a risk of erroneous concluding that the RG philosophy leads to better effects, while any such effect could also be attributed to the differences resulting from the provision of structure for involving significant others. The use of qualitative material to interpret the quantitative findings will help to avoid this risk.

## Abbreviations

ACT: Assertive Community Treatment
AUC: area under the curve
BSI: Brief Symptom Inventory
CEA: cost-effectiveness analysis
CONSORT: consolidated standards of reporting trials
CSQ: Client Satisfaction Questionnaire
CUA: cost-utility analysis
CHEERS: consolidated health economic evaluation reporting standards
DEM: demographic information
EE: expressed emotions
EQ-5D-5L: 5-level euro quality of life 5 dimensions
FACT: Flexible Assertive Community Treatment
GAF: global assessment of functioning scale
ICC: intraclass correlation coefficient
ICER: incremental cost effectiveness ratios
IEQ: Involvement Evaluation Questionnaire
I.ROC: Individual Recovery Outcomes Counter
LQLP: Lancashire Quality of Life Profile
MANSA: Manchester Short Assessment of Quality of Life
NEL: Netherlands Empowerment List
OT: optimal treatment
QALY: quality-adjusted life years
RAAS: Revised Adult Attachment Scale
RACT: Resource Group Assertive Community Treatment
RCT: randomized controlled trial
RG: resource group
RG method: resource group method
RG members: resource group members
R-MET: Resourcegroup Model Evaluation Tool
SMI: severe mental illnesses
SOFAS: social and occupational functioning scale
SPSS: statistical package for the social sciences
TiC-P: Trimbos and Institute of Medical Technology Assessment Cost Questionnaire for Psychiatry
VAS: Visual Analogue Scales
VSSS-EU: Verona Service Satisfaction Scale
WHODAS 2.0: World Health Organization Disability Assessment Schedule
